# A prediction model based on platelet parameters, lipid levels, and angiographic characteristics to predict in-stent restenosis in coronary artery disease patients implanted with drug-eluting stents

**DOI:** 10.1186/s12944-021-01553-2

**Published:** 2021-09-29

**Authors:** Min-Tao Gai, Bing Zhu, Xiao-Cui Chen, Fen Liu, Xiang Xie, Xiao-Ming Gao, Xiang Ma, Zhen-Yan Fu, Yi-Tong Ma, Bang-dang Chen

**Affiliations:** 1 State Key Laboratory of Pathogenesis, Prevention and Treatment of High Incidence Diseases in Central Asia, Clinical Medical Research Institute, The First Affiliated Hospital of Xinjiang Medical University, Urumqi, China; 2 Department of Cardiology, First Affiliated Hospital of Xinjiang Medical University, No. 137, Liyushan Road, Urumqi, China; 3 College of Basic Medicine of Xinjiang Medical University, No. 137, Liyushan Road, Urumqi, China

**Keywords:** In-stent restenosis, Coronary heart disease, Percutaneous coronary intervention, Risk factors

## Abstract

**Background:**

The present study was aimed to establish a prediction model for in-stent restenosis (ISR) in subjects who had undergone percutaneous coronary intervention (PCI) with drug-eluting stents (DESs).

**Materials and methods:**

A retrospective cohort study was conducted. From September 2010 to September 2013, we included 968 subjects who had received coronary follow-up angiography after primary PCI. The logistic regression analysis, receiver operator characteristic (ROC) analysis, nomogram analysis, Hosmer–Lemeshow χ^2^ statistic, and calibration curve were applied to build and evaluate the prediction model.

**Results:**

Fifty-six patients (5.79%) occurred ISR. The platelet distribution width (PDW), total cholesterol (TC), systolic blood pressure (SBP), low-density lipoprotein cholesterol (LDL-C), and lesion vessels had significant differences between ISR and non-ISR groups (all *P* < 0.05). And these variables were independently associated with ISR (all *P* < 0.05). Furthermore, they were identified as predictors (all AUC > 0.5 and *P* < 0.05) to establish a prediction model. The prediction model showed a good value of area under curve (AUC) (95%CI): 0.72 (0.64–0.80), and its optimized cut-off was 6.39 with 71% sensitivity and 65% specificity to predict ISR.

**Conclusion:**

The incidence of ISR is 5.79% in CAD patients with DES implantation in the Xinjiang population, China. The prediction model based on PDW, SBP, TC, LDL-C, and lesion vessels was an effective model to predict ISR in CAD patients with DESs implantation.

## Background

Coronary artery disease (CAD) is a high mortality disease in modern society [1, 2], and it is commonly treated with percutaneous coronary intervention (PCI) [3]. However, the occurrence of in-stent restenosis (ISR) increases the risk of stent failure [4]. Although drug-eluting stents (DESs) are widely applied, ISR is still a crucial issue in the treatment of CAD after PCI [5]. Nowadays, the incidence of ISR remains approximately 10%, which may cause the recurrence of ischemic heart disease [4, 6]. Until now, the incidence of ISR in the Xinjiang population, China, was not reported. Additionally, clinic is still lacking an appropriate prediction model to predict ISR [7]. Establishing a novel prediction model for ISR will contribute to individual risk stratification and ISR prevention for CAD patients [8].

ISR has a distinct pathophysiological process that is not merely accelerated atherosclerosis [9]. ISR is usually caused by increased acute vessel injury and the promotion of neointimal hyperplasia [10]. Acute thrombotic occlusion is a high frequent cause of in-stent chronic total occlusion [11], which is implied that platelets are important in ISR. In previous reports, several risk factors were related to ISR, including diabetes, chronic renal insufficiency, bare-metal stents, small coronary artery vessels, a long stent length, and coronary bifurcation lesions [4, 12]. Recently, studies revealed that the increase of C-reactive protein, homocysteine, and stent numbers are risk factors for ISR [13–15]. However, the roles of platelet parameters in ISR were still less reported. Platelet activation is persistent and usually accompanied by morphological changes after PCI. Inhibition of platelet activation has effects on neointimal proliferation and restenosis [16, 17]. Developing a novel prediction model based on platelet parameters, clinical characteristics, lipid levels, and angiographic characteristicsand may contribute to risk stratification and disease prevention for ISR in CAD patients.

Therefore, this study is set out to reveal the incidence of ISR in the Xinjiang population, China, and explore a potential prediction model for ISR in CAD patients with DES implantation.

## Materials and methods

### Participants

Nine hundred sixty-eight subjects who received PCI and coronary follow-up angiography from September 2010 to September 2013 were included. Inclusion criteria: 1) the subjects were diagnosed with CAD and had undergone PCI; 2) the patients had undergone follow-up angiography more than 6 months after PCI; 3) DESs were implanted; 4) the subjects were from the Han and Uyghur populations; 5) the subjects were aged over 18.

The exclusion criteria were as follows: 1) the patients had myocarditis, pericarditis, myocardiopathy, congenital heart disease, or other structural heart diseases; 2) the patients had other comorbidities, including immune system disease, infectious disease, tumors, or hematologic diseases.

### Diagnostic criteria

ISR was defined as a restenosis degree > 50% based on coronary angiography, which included both the original site where the stent was placed and vascular segments adjacent to the stent at a 5-mm distance; if the restenosis degree was < 50%, the ISR was defined as non-ISR [18].

### Data collection

Demographic information, biochemical parameters, clinical and angiographic characteristics were collected. Fasting blood samples collected from peripheral the vessel were obtained to test routine biochemical variables, including platelet parameters of plateletcrit (PCT), MPV, platelet count, platelet distribution width (PDW), lipid parameters of low-density lipoprotein cholesterol (LDL-C), triglyceride (TG), TC, and high-density lipoprotein-cholesterol (HDL-C). The blood samples were evaluated within 2 h. The specific data concerning angiography and the stents included the stenosis location, lesion vessels, the number of stents, stent parameters, and post-balloon dilatation.

### Statistical analyses

To compare the difference between groups in continuous data, independent samples T-test was used between two groups, and one-way analysis of variance (ANOVA) and Student-Newman-Keuls (SNK) test were performed for more than three groups; the data were expressed as the means ± standard deviation (SD). For variables with an abnormal distribution, the Kruskal-Wallis H test was performed. A chi-squared test was used to compare categorical variables, and the data were expressed as percentages. Multivariate logistic regression analyses were conducted with forward stepwise selection, in which *P*-value levels for inclusion and exclusion criteria were set as 0.05 and 0.10, respectively. ROC analysis was performed for each variate associated with ISR, in which AUC > 0.5 and *P* < 0.05 were included in the prediction model. The Hosmer–Lemeshow χ^2^ statistic, calibration curve, and bootstrap for 1000 times were used to examine the prediction model. To further evaluate the discrimination of the model and optimized cut-off, the ROC analysis and Youden index were used. The internal validation was conducted by the bootstrap method. The total risk score for this prediction model was counted as the equation: Y = 0.1480*PDW + 0.1709*TC + 0.2639*LDL + 0.0121*SBP + 0.276*(lesion vessels), and the scales for each variable were assigned according to their corresponding beta-coefficient identified by multivariate logistic regression. Moreover, the risk score for each variable was conducted by nomogram analysis. A *P*-value < 0.05 was statistically significant. SPSS version 22.0 (SPSS Inc., Chicago, IL, USA) was used to perform statistical analyses in this study. The nomogram analysis and calibration curve were performed by R software version 4.0 (R Foundation for Statistical Computing, Vienna, Austria).

## Results

### Clinical and angiographic characteristics between the ISR and non-ISR groups

Of 968 patients included in our study, 56 subjects (5.79%) had ISR after PCI for an average of 16.93 months. Most of the baseline characteristics between the two groups were similar, such as age, sex, blood glucose, current smoking, alcohol consumption, waist circumference, body mass index (BMI), post-balloon dilatation, balloon diameters, balloon lengths, stent diameters, and stent lengths (all *P* > 0.05). However, as Tables 1 and 2 shown, PDW, TC, HDL, LDL-C, SBP, myocardial infarction (MI), lesion vessels, and right coronary artery (RCA) stenosis showed significant differences between the groups (all *P* < 0.05).

**Table 1:** Comparisons of the baseline clinical characteristics between the ISR and non-ISR groups

Parameters	ISR (***n*** = 56)	Non-ISR (***n*** = 912)	t/ *χ*^2^	***P***
Age, year	59.60 ± 10.88	58.95 ± 10.83	−0.43	0.666
Male, n (%)	47 (83.93)	713 (78.18)	1.03	0.309
Smoking, n (%)	33 (58.93)	465 (50.99)	1.33	0.248
Alcohol consumption, n (%)	7 (12.50)	164 (17.98)	1.09	0.296
BMI, kg/m^2^	25.69 ± 3.27	26.20 ± 3.6	0.95	0.341
Waist circumference, cm	92.87 ± 15.12	92.14 ± 13.45	−0.31	0.758
blood glucose, mmol/L	6.34 ± 3.01	6.48 ± 2.94	0.35	0.729
SBP, mmHg	149.08 ± 31.83	137.39 ± 32.85	−2.5	0.013*
DBP, mmHg	99.17 ± 23.46	96.08 ± 25.04	−0.87	0.385
Ejection fraction, %	61.48 ± 7.18	61.02 ± 6.15	0.41	0.686
Medical history				
Hypertension, n (%)	32 (57.14)	429 (47.04)	2.16	0.168
Diabetes, n (%)	12 (21.43)	190 (20.83)	0.01	0.915
MI, n (%)	23 (41.07)	239 (26.21)	5.91	0.015*
Arrhythmia, n (%)	1 (1.79)	60 (6.58)	2.05	0.152
Stroke, n (%)	0 (0.00)	21 (2.3)	1.32	0.251
Laboratory results				
WBC, ×10^9^/L	7.47 ± 2.16	7.28 ± 2.09	−0.65	0.517
MO, %	7.07 ± 2.35	6.72 ± 2.38	−1.35	0.178
Platelet count, ×10^6^/L	214.02 ± 57.76	216.43 ± 62.67	0.28	0.781
MPV, fl	10.28 ± 1.64	10.38 ± 1.44	0.48	0.634
PCT, %	0.23 ± 0.12	0.23 ± 0.09	−0.17	0.868
PDW, %	16.65 ± 2.37	15.78 ± 2.62	−2.41	0.016*
TC, mmol/L	4.77 ± 1.91	4.15 ± 1.56	−2.74	0.006*
TG, mmol/L	3.69 ± 6.28	2.59 ± 4.18	−1.77	0.076
HDL, mmol/L	1.21 ± 1.44	1.01 ± 0.4	−2.71	0.007*
LDL-C, mmol/L	2.82 ± 1.14	2.46 ± 0.9	−2.74	0.006*
ApoA1, g/L	1.50 ± 1.95	1.45 ± 1.71	−0.21	0.835
GSP, mmol/L	6.12 ± 15.25	3.83 ± 9.61	−1.64	0.102
Lipoprotein(a), mmol/L	237.62 ± 221.45	214.31 ± 190.05	−0.81	0.419
Creatine, μmol/L	78.61 ± 26.63	74.32 ± 21.06	−1.44	0.151
Uric acid, μmol/L	312.99 ± 121.06	316.27 ± 102.75	0.23	0.820
Model	5.85 ± 0.80	5.36 ± 0.69	−4.73	0.000*

* *P* < 0.05, compared with non-ISR group. Abbreviations: *ISR* in-stent restenosis, *BMI* body mass index, *SBP* systolic blood pressure, *DBP* diastolic blood pressure, *MI* myocardial infarction, *PDW* platelet distribution width, *LDL-C* low-density lipoprotein cholesterol, *HDL* high density lipoprotein, *TC* total cholesterol, *TG* triglyceride, *PCT* plateletcrit, *MPV* mean platelet volume, *GSP* glycated serum protein

**Table 2:** Characteristics of angiography, balloons, and stents in the ISR and non-ISR groups

Parameters	ISR (***n*** = 56)	Non-ISR (***n*** = 912)	t/*χ*^2^	***P***
LM stenosis, n (%)	6 (11.11)	51 (5.60)	2.79	0.095
LAD stenosis, n (%)	37 (66.07)	592 (64.91)	0.03	0.86
LCX stenosis, n (%)	29 (51.79)	389 (42.65)	1.79	0.181
RCA stenosis, n (%)	37 (66.07)	460 (50.44)	5.16	0.023*
Lesion vessels	2.61 ± 1.39	2.12 ± 1.44	−2.35	0.014*
Stents	0.23 ± 0.57	0.33 ± 0.75	0.99	0.323
Post-balloon dilatation, n (%)	30 (55.56)	446 (50.80)	0.46	0.575
Balloon diameters (mm)	2.77 ± 1.01	2.95 ± 0.80	−1.13	0.261
Balloon lengths (mm)	12.10 ± 5.17	12.17 ± 4.44	−0.088	0.930
Stent diameter (mm)	2.72 ± 0.29	2.85 ± 1.09	−0.855	0.393
Stent length (mm)	26.28 ± 7.93	26.89 ± 7.45	−0.558	0.577

* *P* < 0.05, compared with non-ISR group. Abbreviations: *LM* left main coronary artery, *LAD* left anterior descending coronary artery, *LCX* Left circumflex coronary artery, *RCA* right coronary artery

### Univariate analysis and multivariate analysis for potential predictors

The parameters of PDW, TC, LDL-C, SBP, MI, lesion vessels, and RCA stenosis were associated with ISR in CAD patients (all *P* < 0.05), and their ORs and 95% CIs were shown in Table 3. In addition, the elevated levels of PDW (OR 1.17; 95% CI; 1.05–1.31; *P* = 0.039), LDL-C (OR 1.34; 95% CI 1.02–1.76; *P* = 0.036), TC (OR 1.18; 95% CI; 1.01–1.39; *P* = 0.039), SBP (OR 1.01; 95% CI; 1.01–1.02; *P* = 0.014), lesion vessels (OR 1.32; 95% CI; 1.06–1.64; *P* = 0.012), and MI (OR 2.34; 95% CI; 1.32–4.15; *P* = 0.039) were still significantly associated with ISR after adjusting by TC, HDL, LDL-C, SBP, PDW, incidence of MI, lesion vessels, and RCA stenosis.

**Table 3:** Logistic regression analysis for potential predictors of ISR

	Univariate analysis				Multivariate analysis			
Parameters	OR	(95%CI)	Wald *χ*^2^	***P***	OR	(95%CI)	Wald *χ*^2^	***P***
TC	1.19	(1.05–1.36)	7.08	0.008*	1.18	(1.01–1.39)	4.25	0.039*
HDL	1.40	(1.04–1.88)	4.97	0.026*				
LDL-C	1.44	(1.11–1.87)	7.33	0.007*	1.34	(1.02–1.76)	4.38	0.036*
SBP	1.01	(1.00–1.02)	6.12	0.013*	1.01	(1.01–1.02)	6.08	0.014*
MI	1.96	(1.13–3.41)	5.72	0.017*	2.34	(1.32–4.15)	8.55	0.003*
PDW	1.14	(1.03–1.27)	5.88	0.015*	1.17	(1.05–1.31)	7.85	0.005*
RCA	1.91	(1.08–3.38)	5.01	0.025*				
Lesion vessels	1.27	(1.05–1.53)	5.90	0.015*	1.32	(1.06–1.64)	3.08	0.012*

* *P* < 0.05. Abbreviations: *ISR* in-stent restenosis, *BMI* body mass index, *MI* myocardial infarction, *PDW* platelet distribution width, *LDL-C* low-density lipoprotein cholesterol, *HDL* high density lipoprotein, *TC* total cholesterol, *TG* triglyceride, *RCA* right coronary artery. Adjust: TC, HDL, LDL-C, SBP, MI, PDW, RCA, lesion vessels

### Identifying predictors in the prediction model for ISR

**Fig. 1:**
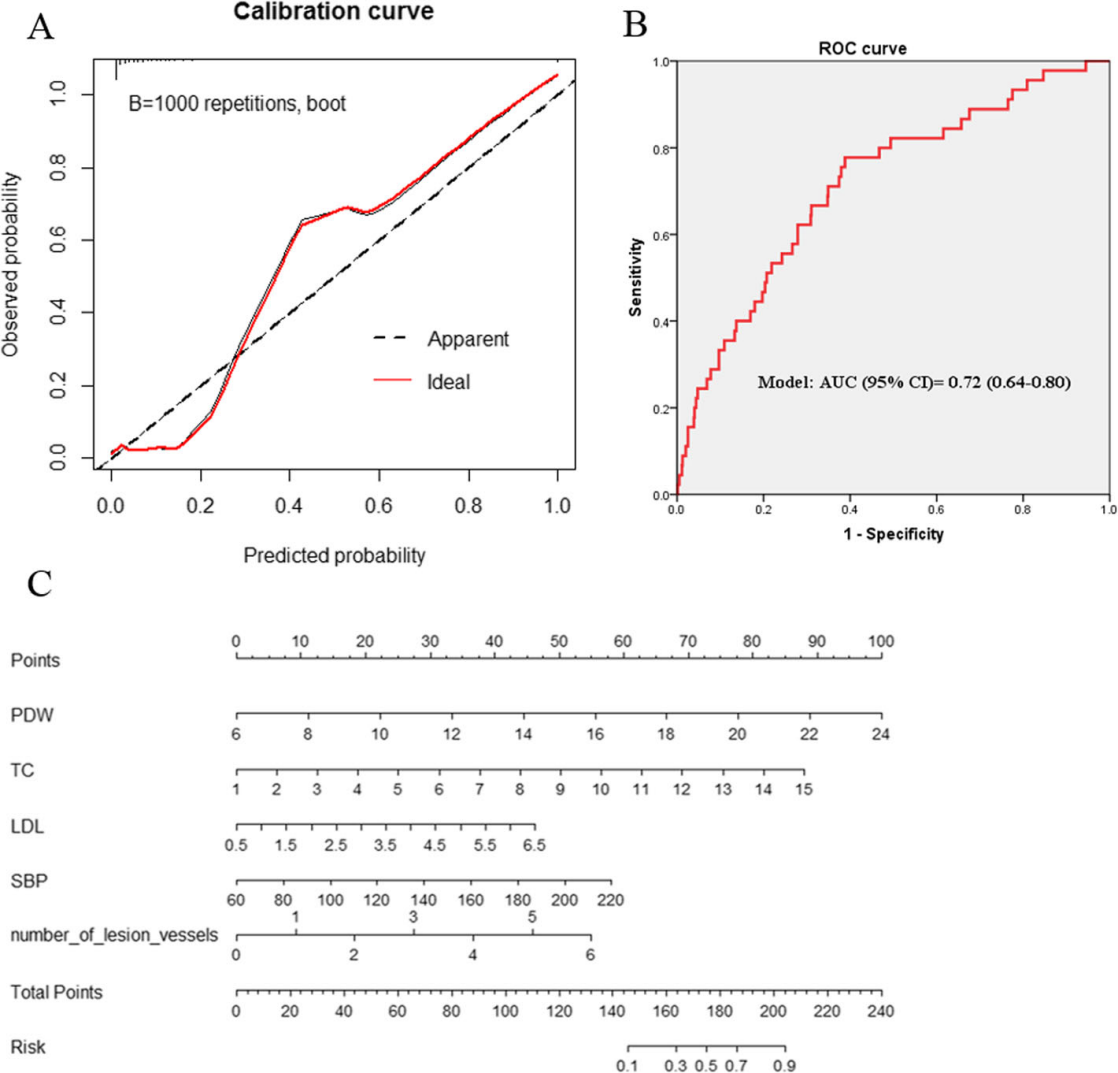
Scoring and evaluating the prediction model. A nomogram was performed for the prediction model. The using instruction for the nomogram is that a line on the top labeled “Points” displays the corresponding score for the values of each variable; a line labeled “Total points”, representing an individual’s total points of all variables in the model, is corresponded to the line labeled “Risk” which indicates the risk of ISR for the individual. Abbreviations: LDL-C, low-density lipoprotein cholesterol; PDW, platelet distribution width; SBP, systolic blood pressure; TC, total cholesterol

To identify potential predictors for ISR, the ROC analyses were conducted for each parameter which independently associated with ISR. At last, the SBP, PDW, TC, LDL-C, and lesion vessels were included in the prediction model, and their AUC and 95% CI were SBP [0.60 (0.51–0.69); *P* = 0.024], PDW [0.60 (0.53–0.67); *P* = 0.042], TC [0.63 (0.54–0.71); *P* = 0.005], and LDL-C [0.61(0.52–0.70); *P* = 0.046], lesion vessels [0.59 (0.52–0.66); *P* = 0.005], respectively (Table 4). To evaluate this prediction model, a Hosmer–Lemeshow goodness-of-fit test was conducted, which yielded *P* = 0.655, and a calibration curve was also displayed in Fig. 1A, indicating no deviation was observed between predicted and observed probability. An internal validation with 1000 times repetitions was performed, and demonstrated a good consistency. In addition, the AUC was 0.72(0.64–0.80) for the prediction model, *P* = 0.000, Fig. 1B.

**Table 4:** ROC analysis for potential predictors of ISR

Parameters	AUC (95% CI)	***P***
SBP	0.60 (0.51–0.69)	0.024*
PDW	0.60 (0.53–0.67)	0.042*
TC	0.63 (0.54–0.71)	0.005*
HDL	0.47 (0.38–0.55)	0.448
LDL-C	0.61 (0.52–0.70)	0.046*
RCA stenosis	0.56 (0.48–0.65)	0.172
Lesion vessels	0.59 (0.52–0.66)	0.030*
MI	0.54 (0.45–0.63)	0.400

*: *P* < 0.05. Abbreviations: *ISR* in-stent restenosis, *SBP* systolic blood pressure, *PDW* platelet distribution width, *LDL-C* low-density lipoprotein cholesterol, *HDL* high density lipoprotein, *TC* total cholesterol, *RCA* right coronary artery, *MI* myocardial infarction

### Nomogram prediction for ISR

The parameters in the prediction model were used to calculate a probability of disease (POD) index which was calculated as the equation Y = 0.1480 ∗ PDW + 0.1709 ∗ TC + 0.2639 ∗ LDL + 0.0121 ∗ SBP + 0.276 ∗ (lesion vessels)A nomogram based on these variables was configured (Fig. 1C). The optimized cut-off for the POD index was 6.39, and its sensitivity and specificity were 71 and 65%, respectively. When the POD index > 6.39, subjects with a high risk of ISR, otherwise with low risk of ISR.

### The association between the prediction model and ISR

As shown in Table 5, the POD index was divided as tertiles, compared with tertile 1 (T1), the OR and its 95%CI of ISR were 2.5 (95% CI: 1.02–6.11, *P* = 0.045) for tertile 2 (T2), and 4.95 (95% CI: 2.15–11.39, *P* = 0.000) for tertile 3 (T3).

**Table 5:** The relationship between the tertiles of the POD index and ISR

Parameters	ISR (***n*** = 56)	Non-ISR (***n*** = 912)	OR(95%CI)	***P***
POD index tertiles				
T1 (< 5.18)	7 (12.50)	315 (34.54)	1.00 (reference)	
T2 (≥5.18 & < 5.60)	17 (30.36)	306 (33.55)	2.50 (1.02–6.11)	0.045*
T3 (≥5.60)	32 (57.14)	291 (31.91)	4.95 (2.15–11.39)	0.000*

*: *P* < 0.05 in chi-square test. Abbreviations: *POD* probability of disease, *ISR* in-stent restenosis

### Overview of the prediction model for ISR

**Fig. 2:**
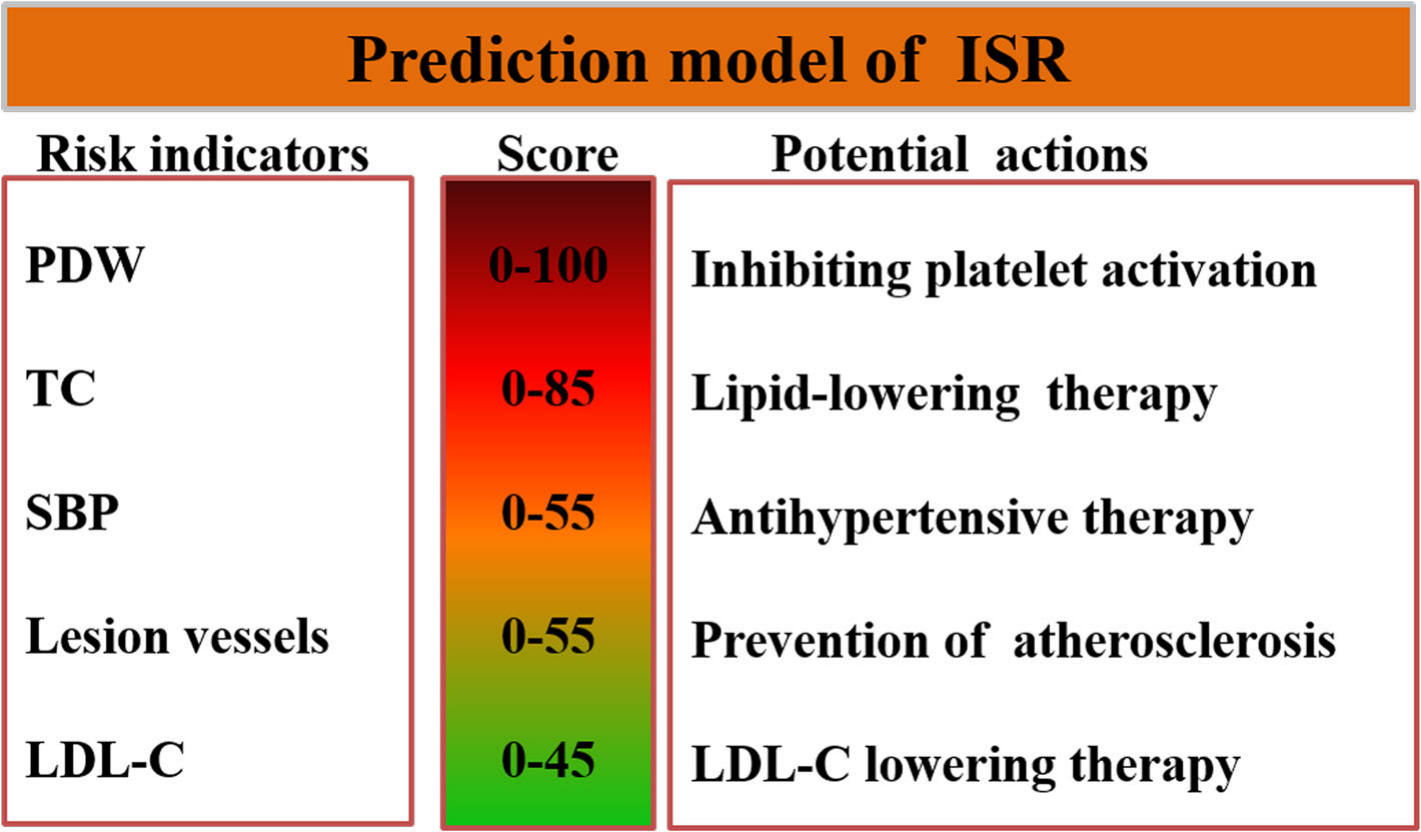
Overview of the prediction model of ISR. The figure summarizes the content of the prediction model, including variables in the model, the lowest and highest risk score for each variable, and suggestions for risk factor control. Abbreviations: LDL, low-density lipoprotein cholesterol; PDW, platelet distribution width; SBP, systolic blood pressure; TC, total cholesterol

As Fig. 2 shown, the overview of the prediction model was indicated, which ranking the predictors as risk scores and presenting actionable preventing methods for each variable.

## Discussion

An effective prediction model can provide useful references for disease prevention. Here, the incidence of ISR in CAD patients with DESs was revealed in the Xinjiang population, China. And a novel prediction model based on platelet parameters, and combined with clinical characteristics and angiographic results was established to predict ISR in patients with CAD and DESs implantation.

Platelet activation plays a crucial role in stent-thrombosis [19] which increased the risk of restenosis [11]. Some studies have explored the relationship between platelet parameter MPV and ISR, but conclusions were inconsistent. A study indicated that MPV is a predictor for ISR in Chinese subjects stented in the carotid artery [20]. While a European study showed that MPV was not a risk factor for ISR after stenting in Whites [21]. The differences may attribute to ethnic differences, for Asians have more prevalent diabetes mellitus, which tends to have higher MPV or clopidogrel resistance [22, 23]. Another platelet parameter PDW is an indicator that reflects the dispersion of the mean platelet volume (MPV), indicates platelet activation [24], and provides more information than MPV [25–27]. The raised PDW indicates an increasing platelet heterogeneity and the occurring of platelet size disparity. And PDW has been reported that associated with the severity and prognosis of CAD [28–30]. Instead of MPV, PDW is an effective predictor to predict ISR in the present study. And its potential mechanisms might be that platelet activation, indicated by elevated PDW, promotes secreting a plethora of chemokines after PCI, like, CXCL4, CXCL12, and CCL5 et al., which recruiting circulating neutrophils and lymphocytes to the place where vascular injured [31]. The process drives the low-grade inflammatory response and promoting the development of neointimal proliferation, and leads to restenosis progression [16, 17, 31, 32].

Raised circulating LDL-C levels play a vital role in atherosclerotic cardiovascular disease [33], and it affects the stenosis of coronary arteries by accelerating the progression of atherosclerosis [34]. Studies have been reported that elevated LDL-C is an independent predictor [15, 35], and comprised of it in prediction models for ISR might be effective [36]. TC is also an independent predictor for ISR in the present study. It has been indicated as an important component of prediction models for cardiovascular disease and recommended to be used to estimate total cardiovascular disease risks [37, 38].

Angiography examination is widely performed to diagnose CAD and to guide the PCI. In recent years, techniques of intravascular imaging detection, such as intravascular ultrasound (IVUS), are gradually applied in PCI. Many studies have revealed intravascular imaging detection has more advantages than angiography, displaying lesion with distinct characterization, and contributing to finding the etiology of ISR, furthermore, using it in PCI procedure decreases the major adverse cardiovascular events for ISR [39–42]. In this study, all PCI guidance or coronary artery examination was performed by the angiography, which may provide less information about lesion and miss some potential predictors. In further studies, establishing a prediction model combined with the lesion characters detected by intravascular imaging might be much valuable.

### Comparisons with other studies and what does the current work add to the existing knowledge

The rates of ISR are varied from study to study in different patients or types of stents. The incidence of ISR is approximately 30% in patients implanted with bare-metal stenting [12]. The application of DESs has significantly reduced the incidence of ISR to 5–15%. While in subjects with diabetes or other comorbidities, the incidence is more than 20% although the DES is applied [25, 43, 44]. In line with previous studies, the incidence of ISR is 5.79% in the present study [4, 6, 14].

A recent study found that a prediction model including abnormal platelets could predict ISR [45]. And another study proved that PDW could predict ISR in patients with CAD and diabetes, which is similar to the present study but with different patients and small sample size [25]. Several prediction models have indicated that models combined with angiographic characteristics, like bifurcation lesion or ≥ 2 vessel-coronary diseases, have good predictive values for ISR [36, 45, 46]. The multivessel lesion has been regarded as a high-risk characteristic of cardiovascular disease. The present study showed that a prediction model comprised of lesion vessels was an independent predictor for ISR, which is similar to previous studies [45]. Beyond characteristics of vessel lesion, pre-procedural and intra-procedural operations of PCI may also influence outcomes of ISR, because various stenting strategies may have different extent impairments to vessels. Therefore, access sites of PCI might also affect the outcomes of ISR for it increasing incidences of adverse events of angiography [47]*.*

### Study strengths and limitations

Here, the present study revealed that the incidence of ISR was 5.79% in CAD patients with DES implantation in the Xinjiang population, China. A novel prediction model based on platelet parameters, and combined with clinical characteristics and angiographic results was effectively to predict ISR in patients with CAD and DESs implantation. Furthermore, a nomogram was provided to evaluate the risk of ISR, and an overview table was listed with potential treatment strategies for CAD patients in clinical practice. Despite these findings, some limitations in this study can not be neglected. First, the study had a retrospective design. Second, only the angiographic examination was applied, the intravascular imaging detections were not performed, which limited the detection of potential predictors and the identification of ISR. Third, the prediction model included the variable of vessel lesions detected by angiography, this might limit the applied range of the prediction model. Besides, this was a single-center study, lacking an external verification. Although an internal validation was conducted, the generalizability of the prediction model may decline.

## Conclusion

In summary, the incidence of ISR is 5.79% in CAD patients with DES implantation in the Xinjiang population, China. The prediction model based on PDW, TC, LDL-C, SBP, and lesion vessels was an effective model to predict ISR, to conduct risk stratification, and prevent ISR in CAD patients with DESs implantation. In further studies, establishing a prediction model combined with more lesion characters detected by intravascular imaging might be much valuable.

## Data Availability

The datasets used and/or analysed during the current study are available from the corresponding author on reasonable request.

## Abbreviations

ANOVA: One-way analysis of variance
BMI: Body mass index
AUC: Area under curve
DESs: Drug-eluting stents
HDL: High-density lipoprotein cholesterol
MPV: Mean platelet volume
PCI: Percutaneous coronary intervention
ISR: In-stent restenosis
LDL-C: Low-density lipoprotein cholesterol
ROC: Receiver operator characteristic
PDW: Platelet distribution width
POD: Probability of disease
PCT: Plateletcrit
SBP: Systolic blood pressure
IVUS: Intravascular ultrasound
MI: Myocardial infarction
TC: Total cholesterol
SD: Standard deviation
RCA: Right coronary artery
TG: Triglyceride
SNK: Student-Newman-Keuls
MI: Myocardial infarction
RCA: Right coronary artery
OCT: optical coherence tomography
